# Supplementary material for: Dynamic causal modelling of COVID-19
Source: Wellcome Open Res. 2020 Aug 7;5:89. Originally published 2020 May 7. [Version 2] doi: 10.12688/wellcomeopenres.15881.2 (PMC7431977; doi:10.12688/wellcomeopenres.15881.2)
Supplement: Peer review history

# Peer review: Reviewer response for version 2

**DOI:** 10.21956/wellcomeopenres.17779.r39871

**Published:** 2020-08-17

**Authors:** Joel Hellewell

**Affiliations:** 1 Centre for the Mathematical Modelling of Infectious Diseases, Department of Infectious Disease Epidemiology, London School of Hygiene & Tropical Medicine, London, UK

I'm very happy with the changes made in the revised version of the paper.

 I personally still struggle with the idea of being able to infer the size of the effective population and what this means conceptually. However, I think the effective population parameter is now adequately explained in the manuscript and is more something for me to think about over the long term rather than something that should prevent indexing.

 I think the authors engaged with my feedback and have responded to what I said, either by expanding the manuscript or showing how such work has been undertaken elsewhere.

Is the rationale for developing the new method (or application) clearly explained?

Yes

Is the description of the method technically sound?

Yes

Are the conclusions about the method and its performance adequately supported by the findings presented in the article?

No

If any results are presented, are all the source data underlying the results available to ensure full reproducibility?

Yes

Are sufficient details provided to allow replication of the method development and its use by others?

Yes

Reviewer Expertise:

Infectious disease modelling, infectious disease epidemiology

I confirm that I have read this submission and believe that I have an appropriate level of expertise to confirm that it is of an acceptable scientific standard.

# Peer review: Reviewer response for version 1

**DOI:** 10.21956/wellcomeopenres.17418.r38865

**Published:** 2020-07-06

**Authors:** Gerardo Chowell

**Affiliations:** 1 Department of Population Health Sciences, School of Public Health, Georgia State University, Atlanta, GA, USA

This technical report presents a dynamical causal model of the transmission dynamics of COVID-19. I believe this paper is one of very few (if any) that follow this type of approach which makes it interesting and an important contribution to the literature even after dozens of modeling papers on the topic have been published or are in the process of publication.

 The paper is well described and the results are interesting and present a new approach for assessing the role of multiple factors on the spread of COVID-19. However, the epidemic has advanced significantly, and it would be good to see how the results and perspectives are shaped by more recent data. Authors should consider updating the paper with the most recent data available, and discuss how their analysis/conclusions are shaped by integrating additional data.

Is the rationale for developing the new method (or application) clearly explained?

Yes

Is the description of the method technically sound?

Yes

Are the conclusions about the method and its performance adequately supported by the findings presented in the article?

Yes

If any results are presented, are all the source data underlying the results available to ensure full reproducibility?

Yes

Are sufficient details provided to allow replication of the method development and its use by others?

Yes

Reviewer Expertise:

Mathematical epidemiology.

I confirm that I have read this submission and believe that I have an appropriate level of expertise to confirm that it is of an acceptable scientific standard.

# Response

**Published:** 2020-07-31

**Authors:** Adeel Razi

**Affiliations:** Monash University, Australia

We would like to thank you Dr. Chowell, for reviewing our manuscript and finding our approach interesting. We have now revised the paper, based on your and another reviewer’s feedback, which is now available.

We have tried to revise the paper to preserve its original content (by limiting changes to the main text to clarify and unpack things). We have used new footnotes no. 11, 13, 16, 24, 31, and 33 and a new section “Posthoc evaluation of model predictions” to address issues that have arisen since submission (for example, the validity of predictions in light of actual outcomes).

We hope this revised version is helpful in further clarifying our new approach.

# Peer review: Reviewer response for version 1

**DOI:** 10.21956/wellcomeopenres.17418.r38986

**Published:** 2020-06-18

**Authors:** Joel Hellewell

**Affiliations:** 1 Centre for the Mathematical Modelling of Infectious Diseases, Department of Infectious Disease Epidemiology, London School of Hygiene & Tropical Medicine, London, UK

This is an interesting and expansive modelling paper from a group of scientists that do not primarily focus on modelling infectious diseases, I think contributions to epidemiology from other fields should always be welcomed and this is no exception. The techniques employed in this paper are less of a different type of model and more of an entirely different modelling framework. As such, I see part of my job in this review as trying to bridge the gaps between the language and techniques of dynamic causal modelling and infectious disease modelling. Hopefully in doing so I am able to present any criticism in a way that both the authors and other infectious disease modellers are able to follow and understand.

 The dynamic causal model developed in this paper can be understood roughly as a stochastic compartmental SEIR model that has 1) a “generative” model that describes movement between unobserved states over time (infection, recovery etc) and 2) an “observational model” that describes the likelihood for the parameter values in the generative model given the observed data (in this case daily deaths and positive tests). The generative model has four components: location, which determines where you are and the contacts you make; infection, which is akin to the susceptible - exposed - infectious - recovered model used commonly; clinical, which determines the clinical presentation should you become infected; testing, which links your current infection status to the result of a swab test. You can be at various states within any of these four components at once, for example I could be an asymptomatic, infectious person at work that has not been tested. How I move between these states is governed by a matrix of probabilities that can be non-linear in time and as a response to feedback from other parameters within the model (for example my probability of observing lockdown can grow as more people die during the outbreak).

 I think ultimately the generative model is comparable to a complicated SEIR model and the next step in the mind of an infectious disease modeller is to use the likelihood from the observational model in a fitting method such as MCMC to generate samples from the posterior distribution of the generative model parameters. Instead, dynamic causal modelling has a developed body of theory that allows for approximation of the analytical solution to the posterior of the model parameters that maximises the model evidence (marginal likelihood). This allows for immediate comparison of different generative model structures on the same data through selecting the model with the optimal log model evidence, which is also referred to as “variational free energy” (a similar process to the commonly used Akaike or Bayesian information criterion). This was refreshing to me as it can sometimes be difficult to obtain AIC/BIC after fitting your model depending on how you have fit it, such as in the probabilistic modelling language Stan where you sometimes need to calculate the leave-one-out information criterion (LOO-IC) yourself.

 Another interesting methodological addition from the dynamic causal modelling framework is fitting the model to data from several different countries and then assigning model parameters as fixed or random effects, using a generalised linear model to estimate the between-country effects of certain covariates. In the manuscript the authors show the results of this process, finding a relationship between the latitude of a country and the effective population size of the outbreak inferred by the model. While, as the authors acknowledge, latitude here is very likely a proxy for other socio-economic variables, this approach could potentially yield interesting results using a wider selection of between-country effects or as a heuristic device to try and understand what factors are driving the model fit in each country. This is complemented with a technique called “Bayesian model reduction”, which efficiently prunes redundant parameters out of the model to simultaneously achieve model parsimony and perform a sensitivity analysis of sorts since it involves fixing the prior of each parameter and looking at the difference in model fit. To me, the framework of dynamic causal modelling seems to make available several tools that should be of interest to infectious disease modellers. It is not the case that infectious disease modellers don’t already try to reduce models or compare them between countries, but what is attractive about the dynamic causal modelling approach is the coherency of the framework and the availability of software to perform the methods for models in general (although I think most infectious disease modellers would prefer to use R rather than MATLAB). At the very least, the methods employed in the dynamic causal modelling framework could be adapted to work with the more familiar combined compartmental model and MCMC approach. The methods in the dynamic causal modelling framework are heavily used and accepted in the field of neuroscience, so I don’t think it’s my job in this review to scrutinise them in particular outside of understanding them to the point where I can understand how the model in this particular paper was fitted. 

 With the general modelling approach summarised I can move on to the specifics of the structure of the generative and observation models:

**Predictive validity**

 In a similar way to AIC/BIC, I think I am correct in thinking that model selection using variational free energy only provides a relative score of model fit and not an objective score. Choosing the best model out of a suite of models does not guarantee that this best model fits well, for this we need to turn to predictive validity and this is where I think the model laid out in the paper is at its weakest. Below is the best-fitting model’s prediction for London in full:

*“Based on current data, reports of new cases in London are expected to peak on April 5, followed by a peak in death rates around April 10 (Good Friday). At this time, critical care unit occupancy should peak, approaching—but not exceeding—capacity, based on current predictions and resource availability. At the peak of death rates, the proportion of people infected (in London) is expected to be about 32%, which should then be surpassed by the proportion of people who are immune at this time. Improvements should be seen by May 8, shortly after the May bank holiday, when social distancing will be relaxed. At this time herd immunity should have risen to about 80%, about 12% of London’s population will have been tested. Just under half of those tested will be positive. By June 12, death rates should have fallen to low levels with over 90% of people being immune and social distancing will no longer be a feature of daily life.”*

 It’s quite hard to tell if we are meant to interpret this as an example of what sort of narrative could be derived from the results of the model, or whether this is a genuine model prediction. If it is the latter, then I would expect to see mention of when the prediction was made, as well as plots showing the prediction (shown in Figures 12 and 6) against the data which is now available. The authors do this for their predictions for Italy (Figure 13) but not London. I am writing this review on June 10th and at the time of writing the number of deaths on the 9th June was 286. Without numbers given for the prediction it’s hard to know if this counts as “low levels” or not, the 8th June was the beginning of week 24 and the corresponding prediction of daily deaths in Figure 12 is near zero. 

**Effective population size**

 Perhaps more concerning than the prediction for deaths is the prediction for immunity. In the paper I find it quite difficult to tell what exactly is being spoken about when it comes to immunity. The model fits a parameter called “effective population” (θN) that I think could do with some further explanation, it seems to be the case that immunity is presented as the number of infections inferred by the model divided by the effective population. When the model was fitted to UK data it inferred an effective population size of ~2.5 million people. It’s quite hard to tell but from Figure 8, looking at the cumulative cases inferred by the model and the proportion of the population entering the immune category, it seems like the model has predicted that nearly all of the 2.5 million people in the effective population are now immune. 

 Here is what the authors say about the effective population parameter:

*“In this technical report, we will choose a simpler option that treats a pandemic as a set of linked point processes that can be modelled as rare events. In other words, we will focus on modelling a single outbreak in a region or city and treat the response of the ‘next city’ as a discrete process post hoc. This simplifies the generative model; in the sense we only have to worry about the ensemble dynamics of the population that comprises one city. A complimentary perspective on this choice is that we are trying to model the first wave of an epidemic as it plays out in the first city to be affected. Any second wave can then be treated as the first wave of another city or region .Under this choice, the population size can be set, a priori, to 1,000,000; noting that a small city comprises (by definition)a hundred thousand people, while a large city can exceed 10 million.*

*Note that this is a prior expectation, the effective population size is estimated from the data: the assumption that the effective population size reflects the total population of a country is a hypothesis (that we will test later).”*

 It is true that you can use a model with a population size under 67 million, look at the dynamics of the outbreak from the model output, and infer things about the potential effectiveness of social distancing, eventual likelihood of herd immunity, and so on, that would be true in a larger population. However, you would not fit a model to death data for all of the UK using a population parameter that is smaller than the population of the UK. I think the model output as shown in the manuscript is a best guess at the outbreak dynamics if the number of deaths and cases observed in a place with a population of 67 million people were instead observed in a place with a population of 2.5 million. As a result of fitting to death rates for a population 30 times bigger than the one in your model, you would expect to find that almost everyone is infected. 

 Since the writing of this manuscript, serological studies have started to emerge which estimate the percentage of the population that have been infected (which would correspond to the immune compartment in the model) . On the 24th May the ONS estimated that around 7% of the UK have antibodies for COVID-19, rising to 17% in London. Even acknowledging that serology studies are not perfect and that the ones performed so far have been quite small scale, this is really quite a different picture than the 90% population immunity presented by the model output.

 The picture is similar in serological studies across the world, even in healthcare workers in hard-hit cities like Barcelona that would have faced constant exposure to infection.

 What is the result of fitting the model to UK deaths and reported cases with a fixed, actual value for the effective population? Or at least using the UK population as the prior value? I think either a) the model output should be more clearly presented as an example or b) you should acknowledge that the model output gives predictions that seem very different from the emerging evidence

**CCU fatality**

 The fitted probability that a person dies given that they are in the CCU (θfat) for China and Italy is very high (nearly 100% and well over 50%). How well does this compare to actual observed mortality rates in CCUs?

 For example, this paper ^1^, found 26% mortality in ICUs in Lombardy, Italy in early March.

**Observation model**

 The UK data collated by the John Hopkins COVID-19 data repository that the authors use fetches data from here.

 The observation model could be improved by including a delay between the actual occurrence of death and its eventual reporting in the official statistics, sometimes it can take a couple of days for deaths to appear in the government figures. I think this could interfere with the model fit as it tries to align deaths and reported cases (which it currently reasons have both happened on that day).

**Reporting structure**

 It is also important to consider the structure of the surveillance system when trying to fit to reported cases. In the UK for a good while tests were only undertaken on hospital admissions that were severe enough to warrant being admitted overnight (or at least that is what the official policy was). Other countries like South Korea had drive-through test centres. This is going to cause a huge discrepancy in how you should interpret changes in reported cases.

**Between-country parameter value variation**

 It is strange that there is so much variation in some of the parameters between countries. For example, the contagious period is around 1 day in China but around 3-5 days in France? What is the biological reasoning behind this? Arguably there could be some genetic variation in the virus between countries but could that cause such a significant difference? Is there any empirical evidence that supports differences in how long your are contagious between countries?

 The same goes for the numbers of contacts at home or contacts at work. People in the United Kingdom are estimated to have around 7 contacts at home, but the average size of UK households is just 2.3. It would be good to link the output of these variables to any empirical data that is available to show that they are meaningful and do actually correspond to whatever data might be available. One of the countries with the lowest effective contacts in the household (~ 1.5) has a higher average household size than the UK of 2.5.

 The variable for the probability of infection given contact (θtrn) is fairly stable apart from China and Australia where it is relatively large and small, respectively. Do the authors have any thoughts why this might be the case? 

**Age structure**

 The model does not include any kind of age structure. Age has a large effect on the fatality of infection and should therefore be accounted for. Countries with an older population would likely see a higher fatality rate. Age could also influence the amount and types of contacts that people make, with more intergenerational contacts happening within the home and more intragenerational contacts happening at work or school. 

**Summary**

 The model described in this paper is an interesting and important first step at putting together a model of infectious disease dynamics within the framework of dynamic causal modelling. However, when the particular model here is fit to data I don’t think it displays that it has captured the dynamics of the outbreak well wherever it is able to be compared to separately collected bits of data such as seroprevalence or CCU mortality. 

 I think what has happened in the model fitting process for the most part is that the variation introduced into the time series of deaths and reported cases due to differing surveillance and reporting structures, differing testing regimes, differing outbreak responses, and differing population demographics between countries have been accounted for within the generative model through between-country variation in parameters such as the effective population size, numbers of contacts at work (for example, do most people in China really have between 100 and 150 effective contacts at work?), CCU fatality, contagious period length, and others. The unfortunate reality is that with a flexible enough model (in terms of numbers of parameters) it is always possible to produce a fit that very closely matches the reported case and death data observed so far. The real test for this model is whether the estimated parameter values that can be compared to other sources of data match what we observe empirically and I think it is fairly obvious that this has not happened. 

 Sadly I don’t think that I can recommend this paper for indexing as it currently stands because I don’t think it is clear what it is trying to do. I think the easiest way of resolving this problem is for the authors to ask themselves the question “Do I think the model predictions made for the UK in this paper are plausible or are they examples of predictions that can be made from the model?”. If the predictions are examples then this paper is an introduction to disease modelling using dynamic causal modelling and the predictions should be more clearly labelled as examples. The paper could then be further improved by showing how methods such as the between-country parameter comparisons using the hierarchical GLM correspond to the types of questions that disease modellers want to answer. Alternatively, if the authors do think that the predictions made in this paper are accurate, then they need to be far more stringent comparing their predictions with data that has become available since they are made and have questions to answer regarding the gap between the 90% immunity in London that they predict and the 17% that has been estimated by the ONS. That London may have already reached herd immunity has huge implications for future intervention policies, the most significant being that there is no danger of a second wave. If we behave as if there is 90% immunity (completely end social distancing etc.) but we are in fact well below herd immunity, then we will have likely caused the second wave through our own actions.

**Recommendations in brief:**

- Compare the prediction made in the manuscript to observed deaths and cases that have happened since.
- Compare the model output used to make the prediction to seroprevalence surveys and evaluate the predictive validity of the model on this.
- Explain what is meant by effective population size and show what happens when predictions are generated using an effective population size equal to the population of the United Kingdom.
- Consider improvements to the observational model that account for reporting delays in death data (and potentially in confirmed cases too, since in the UK they are likely people that were tested due to hospital admission and therefore there is a period of time between onset and hospital admission).
- Try to compare the posterior estimates of other model parameters (household contacts etc) to existing data. The usual approach would be to fix the parameter values in the model using existing data and then fitting the unknown parameters. This model fits a lot of parameters including some for which there is existing data. If the model fits parameter values that differ from the existing data, this needs to be explained by the authors.

Is the rationale for developing the new method (or application) clearly explained?

Yes

Is the description of the method technically sound?

Yes

Are the conclusions about the method and its performance adequately supported by the findings presented in the article?

No

If any results are presented, are all the source data underlying the results available to ensure full reproducibility?

Yes

Are sufficient details provided to allow replication of the method development and its use by others?

Yes

Reviewer Expertise:

Infectious disease modelling, infectious disease epidemiology

I confirm that I have read this submission and believe that I have an appropriate level of expertise to state that I do not consider it to be of an acceptable scientific standard, for reasons outlined above.

## References

[1] : Baseline Characteristics and Outcomes of 1591 Patients Infected With SARS-CoV-2 Admitted to ICUs of the Lombardy Region, Italy. *JAMA*.2020;323(16) : 10.1001/jama.2020.5394 10.1001/jama.2020.5394 PMC713685532250385

# Response

**Published:** 2020-07-23

**Authors:** Adeel Razi

**Affiliations:** Monash University, Australia

Dear Dr. Hellewell,

We would like to thank you for the considerable time and effort you have spent reviewing our manuscript. Your thoroughness and attention to detail, in what must be very busy and challenging times, has been very much appreciated. We were particularly impressed with the summary of the technical aspects of this work, which are useful and informed descriptions in their own right.

We have tried to revise the paper to preserve its original content (by limiting changes to the main text to clarify and unpack things). We have used footnotes and a new 'to address issues that have arisen since submission (for example, the validity of predictions in light of actual outcomes).

Below are the replies to the comments, that for clarity we have grouped into key themes. We hope these revisions are what you had in mind:

**A) Predictive validity:**

The primary purpose of this paper was to serve as a technical report, introducing a methodology that could be, and was, used to answer specific questions about epidemiological parameters and epidemiological model structure. To clarify this, we have emphasised that the narrative at the end of the paper is an example of the kind of predictions that can be made, rather than a definitive prediction *per se *(footnote 31):

*“This narrative is not offered as a prediction – but as an example of the kind of predictions afforded by dynamic causal modelling. An aspect of these predictions is that they include systemic factors beyond the epidemiology per se. The best example of this is the above predictions about social distancing, which could be read as ‘lockdown’; namely the probability that I will leave home. This highlights a key distinction between dynamic causal models and standard quantitative epidemiological modelling that treats things like ‘lockdown’ as interventions that are supplied to the model. In contrast, interventions such as social distancing and testing are modelled as an integral part of the process – and are estimated on the basis of the data at hand. One consequence of this is that one can make predictions about when ‘interventions’ – or their suspension – will occur in the future.”*

Regarding specific predictive validity, we thought it would be disingenuous to change the predictions in light of subsequent outcomes—or the procedures that were applied in subsequent reports. However, we have now added an extensive ‘Posthoc evaluation of model predictions’ section in the revised version that addresses the predictions in light of current data. This section implicitly addresses the specific points about predictions in the reviewers’ comments. We have also attempted to make the demarcation between a procedural and predictive contribution clearer throughout the text by including footnotes like the following (footnote 33):

*“To reiterate, the purpose of this technical report was to introduce the variational procedures entailed by dynamic causal modelling in the setting of quantitative, epidemiological modelling. Since this report was submitted, several papers have used procedures described in this report to address specific questions; for example, the impact of lockdown cycles, the effect of population fluxes among regional outbreaks, the efficacy of testing and tracing, and the impact of heterogeneous susceptibility and transmission. Crucially, in line with a key message of this foundational paper, each successive application of the dynamic causal modelling leveraged Bayesian model comparison to update the model as new data became available.”*

We also take the opportunity to future-proof retrospective evaluations of the reproduction ratio with the following footnote 13:

*“Added in revision: the reproduction ratio in this report was based upon an approximation to the expected number of people that I might infect, if I was infectious. In subsequent reports, the reproduction ratio was brought into line with more formal definitions, based on the geometric rate of increase in the prevalence of infection and the period of contagion. A minimum reproduction ratio (R) of nearly zero in this report corresponds to about 0.7 in subsequent (and other) reports.”*

In addition to these, we have also incorporated a number of additional changes outlined below. 

**B) Effective population**

It is clear that the “ *effective population*” terminology, particularly in respect to immunity, represents a common source of confusion. To rectify this, we have made a number of changes throughout the paper. First, we have amended the “Initial Conditions and population size” section, splitting it and introducing a new subsection as follows: 

***“Effective Population*** *:*

*Under the initial conditions, the population size can be set, a priori, to 1,000,000; noting that a small city comprises (by definition) a hundred thousand people, while a large city can exceed 10 million. This population parameter is a prior that is updated based on the available data, providing an estimate of the “effective population” size. Effective population is defined here as the proportion of the total population who are susceptible to infection, and therefore participate in the outbreak. The assumption that the effective population size reflects the total population of a country is a hypothesis that we will test later [footnote 16]. For clarity, we are not implying that the remainder of the population classed as “not susceptible” are immune or resistant to COVID-19, rather there exists a sub-population who do not take part in the current outbreak for any of a variety of reasons that may include being shielded or geographically isolated from infected cases. Furthermore, as the effective population (and other parameters) are estimated directly from the data, they will therefore reflect the source of the information. At the time of writing, in the UK this was dominated by the London outbreak. Finally, as all parameters pertain to the effective population, proportions (or probabilities)—such as population immunity—require appropriate scaling to be expressed as a percentage of the total (census) population.”*

*[Footnote 16]:This technical report considered an outbreak in a single region or city. A country-wide (pandemic) model of viral spread would require multiple regional models to be coupled together, and is the focus of subsequent papers (K. J. Friston, T. Parr, P. Zeidman, A. Razi, G. Flandin, J. Daunizeau, O. J. Hulme, A. J. Billig, V. Litvak, C. J. Price, R. J. Moran, C. Lambert, (2020) ‘‘Second waves, social distancing, and the spread of COVID-19 across America’’, arXiv: :2004.13017).*

We have also annotated the legend to figure 11, and made the following change to immunity predictions, to clarify this further:

*“Note that predictions—like the percentage of herd immunity—pertain to the effective population. For example, if 80% of the effective (2.5 million) population are seropositive, one would expect 22% of the census (8.9 million) population of London to have seroconverted by early May.”*

**C) Improvements to model:** 

We appreciate the number of suggestions to help refine or improve this model further. As surmised in the “Predictive Validity” section of your review, this report provides an initial technical description for the kind of analyses that could be used via the presented methodology. In a sense, it represents a proof of concept for this type of modelling, and we acknowledge there are many directions and improvements that could be made such as regional specific models (e.g. https://www.fil.ion.ucl.ac.uk/spm/covid-19/dashboard/local/), or integrating demographic data for known risk factors (e.g. age, ethnicity, BMI etc.,). To reflect this, we have amended the conclusion as follows:

*“This technical report describes an initial implementation of the DCM framework to provide a generative model of a viral epidemic, and to demonstrate the potential utility of such modelling. Clearly there are a number of ways this model could be refined. Our hope in making it open source is that it will allow others to identify issues, contribute to improvements—and help facilitate objective comparisons with other models—using Bayesian model comparison.*

*There remain a number of outstanding issues:* *”*

Additionally, in a separate piece of work [1] we have also formally compared an ODE-based SEIR model to the DCM presented here. Here the SEIR was developed originally by Moghadas et al. [2] to assess CCU projections due to COVID-19 in the US. The SEIR model comprised 12 states including asymptomatic and subclinical infected states, self-isolation, and separate states of hospitalization [2]. We optimised parameters for both the SEIR and DCM using identical variational processes to those presented here. Taking data from seven European countries including the UK, we found that the approximate model evidence or Free Energy provided very strong support for the DCM as compared to the SEIR model, suggesting that marginal state occupancy was important when accounting for those data. In particular Log Bayes Factors of >100 was evidenced for all seven datasets. This comparative analysis is currently under review.

**D) Observation model**

We thank the reviewer for highlighting this. We are aware that delays in reporting deaths and reporting of statistics over weekends do represent potential confounds to the observed time series data. In this work, we perform smoothing of time series by several days to deal with these delays in reporting. Delays in reporting PCR testing were modelled explicitly in terms of a ‘waiting for a test’ state because entry into this state depends upon testing capacity. Conversely, a simple delay in reporting a death can be accommodated by an increase in effective dwell time in critical care. One could consider a DCM that modelled the delay in reporting deaths explicitly—and then use Bayesian model comparison to compare models with and without delays. We did not do this; however, the conditional dependencies between an additional delay parameter and the existing parameters would probably reduce the marginal likelihood (i.e., Bayesian model evidence) of an extended DCM.

**E)** **Reporting structure**

We agree that differences in testing and reporting strategies will impact the data. In the model presented, the testing rate parameter accounts for some of these differences. We have added the following footnote 11 to emphasise the importance of this part of the model.

*“It is revealing to note that the number of model parameters pertaining to PCR testing matches the number of parameters mediating the epidemiology per se. This reflects the fact that the generative model has to consider every aspect of how data are generated. In order to leverage the information in new positive tests, it is necessary to think carefully about all the parameters that contribute to these data; for example, the probability of being tested and the selection bias towards testing people who are more likely to be infected. Crucially, this bias has to be estimated during model inversion and could vary substantially from country to country. Although not implemented in this report, subsequent distinctions between Pillar 1 and 2 test data would be a nice example of different selection biases. This speaks to the importance of modelling Pillar 1 and 2 as distinct data modalities. From a technical perspective, equipping standard epidemiological models with an ‘observation model’ can be regarded as building a complete dynamic causal model. The key thing to bear in mind here is that the parameters of so-called observation models have to be treated in exactly the same way as epidemiological parameters, because they could show conditional dependencies. In dynamic causal modelling, all unknown parameters are treated in a uniform way to maximise (a free energy bound on) marginal likelihood.”*

**F) CCU fatality/Between-country parameter value variation**

First a disclaimer is that these assertions (for example Fig. 6, showing differences among countries) are not about actual states of affairs. These are the best explanations for the data available at the time, under the simplest model of how those data were caused. However, there does appear to be some degree of predictive validity; for example, the predicted CCU mortality rate in the UK in April (at the time of writing of the paper) of about 48%, was close to data published on the 4th April by the Intensive Care National Audit and Research Centre (critical care mortality = 50.1% [3]). Regarding the Italian data from Lombardy, whilst the mortality rate was lower (26%), the data was acquired earlier on in the pandemic (February 20 to March 18) before the peak in cases. Rather than dissect the predictive validity of each parameter and country, which is widely recognised as an extremely challenging problem [4], we would reiterate that this paper is intended as a technical report for DCM, and provides examples of the types of questions that could be addressed using this method. To clarify these points, we have modified the following in the “Parametric empirical Bayes and hierarchical modelling section”:

*“Again, these assertions are not about actual states of affairs. These are the best explanations for the data available at the time, under the simplest model of how those data were caused [footnote 24].*

*[Footnote 24]: However, there does appear to be some predictive validity to these that are addressed in a '*Posthoc evaluation of model predictions' section *. Note rather than dissect the predictive validity of each parameter and country, which is widely recognised as a challenging problem (2), we have provided some representative examples. A comprehensive analysis of this type would be beyond the scope of this report. It is also important to note that predictions based upon rate parameters and probabilities are a reflection of prior assumptions about these parameters, whereas predictions based upon the hidden states speak to the predictive validity of the DCM model structure (see below).”*

We have also updated the London population (previously mentioned as 7 million) which is 8.96 million (mid-2019 estimate) [5].  

**References**

[1] Moran RJ, Fagerholm ED, Cullen M, Daunizeau J, Richardson MP, Williams S, et al. Estimating required'lockdown'cycles before immunity to SARS-CoV-2: model-based analyses of susceptible population sizes,'S0', in seven European countries, including the UK and Ireland [version 1; peer review: awaiting peer review]. Wellcome Open Research. 2020;5.

[2] Moghadas SM, Shoukat A, Fitzpatrick MC, Wells CR, Sah P, Pandey A, et al. Projecting hospital utilization during the COVID-19 outbreaks in the United States. Proceedings of the National Academy of Sciences. 2020.

[3] www.icnarc.org/DataServices/Attachments/Download/76a7364b-4b76-ea11-9124-00505601089b

[4] Phua J, Weng L, Ling L, Egi M, Lim CM, Divatia JV, Shrestha BR, Arabi YM, Ng J, Gomersall CD, Nishimura M. Intensive care management of coronavirus disease 2019 (COVID-19): challenges and recommendations. The Lancet Respiratory Medicine. 2020 Apr 6.

[5] https://en.wikipedia.org/wiki/Greater_London

******************************************************************************************************************** 

Posthoc evaluation of model predictions

This section was written three months after the report was submitted, providing an opportunity to revisit some of the predictions in light of actual outcomes. Although the predictions in this report were used to illustrate the nature of the predictions supported by models that included social distancing, they can be used to assess the predictive validity of the DCM.

Subsequently, the DCM was optimized using Bayesian model comparison. A crucial addition was the inclusion of heterogeneity in the response of the population to viral infection. However, even the simple DCM above accommodated sufficient heterogeneity—in terms of the distinction between an effective and total (census) population—to provide some accurate predictions.

In brief, the shape and timing of the epidemic in London was predicted to within a few days. Conversely, the number of fatalities and positive test results were overestimated by a factor of about 3. In what follows, we list the accurate and inaccurate predictions. We assume that the census population of London was 8.96 million [1]. *London's population* is taken to be the effective population estimated to be 2.49 million (see Table 2) and *social distancing* is read as lockdown (i.e., the probability of leaving home).

**Accurate predictions**

 

- “ *Based on current data, reports of new cases in London are expected to peak on April 5*”

Daily confirmed cases of coronavirus in the UK peaked on April 5 [2]

 

- “ *A peak in death rates around April 10 (Good Friday).*”

Peak death rates in London reached 249 per day on April 9 [3]

 

- “ *At this time *[April 10] *, critical care unit occupancy should peak, approaching—but not exceeding—capacity*”

During the Easter weekend of 11–12 April, the NHS Nightingale Hospital London had only 19 patients. Existing London hospitals had sufficient capacity after increasing their combined intensive care capacity from 770 beds to 1,555. As of April 24, only 41 patients had been treated at the Nightingale hospital [4].

 

- “ *At the peak of death rates *[April 10] *, the proportion of people infected (in London) is expected to be about 32%*”

This prediction corresponds to 8.9% = 32% x 2.49/8.96 of the census population of London, which coincides with the consensus estimates at that time. “Professor Chris Whitty admits he thinks at least 10% of the capital has been infected” (published on 24-April-2020) [5].

 

- “ *Improvements should be seen by May 8, shortly after the May bank holiday, when social distancing will be relaxed.*”

On May 8, the first Black Lives Matter demonstrations started in London. This was followed by the **first governmental relaxation of lockdown** on May 10: “So, work from home if you can, but you should go to work if you can’t work from home.” (Prime Minister's address to the nation: 10-May-2020) [6]

 

- “ *At this time *[May 8] *herd immunity should have risen to about 80%”*

Population immunity in the effective population corresponds to 80% x 2.49 / 8.9 = 22% seroprevalence in the census population, which had risen to 17.5% in the previous week: “After making adjustments for the accuracy of the assay and the age and gender distribution of the population, the overall adjusted prevalence in London increased from 1.5% in week 13 to 12.3% in weeks 15 to 16 and 17.5% in week 18” (week ending May 3, 2020) [7].

 

- “ *By June 12, death rates should have fallen to low levels with over 90% of people being immune*”

Weekly reported deaths in London hospitals for the week ending June 11 fell to 22 (with positive tests)[8]. Seroprevalence for this period was not reported.

 

- “ *By June 12, social distancing *[lockdown] *will no longer be a feature of daily life.*”

The **second governmental relaxation of lockdown** was announced on June 10 and June 23, with an initial reopening of shops, and an easing of the two-metre social distancing rule:

“[A]s the Business Secretary confirmed yesterday, we can now allow all shops to reopen from Monday.” (Prime Minister's statement that the coronavirus press conference: 10-June-2020) [9]

“Thanks to our progress, we can now go further and safely ease the lockdown in England.  At every stage, caution will remain our watchword, and each step will be conditional and reversible. Mr Speaker, given the significant fall in the prevalence of the virus, we can change the two-metre social distancing rule, from 4th July.” (Prime Minister's statement to the House: 23-June-2020) [10]

**Inaccurate predictions**

 

- “ *About 12% of London's population will have been tested (May 8). Just under half of those tested will be positive*.”

This was an overestimate: 12% of the effective population corresponds to 143,424 = 12% x .48 x 2.49 positive tests. At the time of writing (17-July-2020), only 34,397 people have tested positive in London [11]—a quarter of the predicted number.

 

- *From Figure 8: Peak daily death rate 807 (710-950) with cumulative deaths of 17,500 (14,000-21,000)*

These were overestimates; daily deaths in London peaked at 249 on April 9 with cumulative deaths at the time of writing (17-July-2020) of 6,106 [12]. This represents consistent overestimates by factors of 3.2 and 2.8, respectively. This may reflect the fact that the data used in the report included regions in the United Kingdom outside London.

[1] https://en.wikipedia.org/wiki/Greater_London

[2] https://www.bbc.co.uk/news/uk-51768274

[3] https://en.wikipedia.org/wiki/COVID-19_pandemic_data

[4] https://en.wikipedia.org/wiki/NHS_Nightingale_Hospital_London#cite_note-HSJ-19.

[5] https://www.dailymail.co.uk/news/article-8250371

[6] https://www.gov.uk/government/speeches/pm-address-to-the-nation-on-coronavirus-10-may-2020

[7] https://www.gov.uk/government/publications/national-covid-19-surveillance-reports/sero-surveillance-of-covid-19

[8] https://www.london.gov.uk/coronavirus/coronavirus-numbers-london

[9] https://www.gov.uk/government/speeches/pm-statement-at-the-coronavirus-press-conference-10-june-2020

[10] https://www.gov.uk/government/speeches/prime-ministers-statement-to-the-house-on-covid-19-23-june-2020

[11] https://coronavirus.data.gov.uk/

[12] https://en.wikipedia.org/wiki/COVID-19_pandemic_data
